# Effects of Genetic Variants of Nuclear Receptor Y on the Risk of Type 2 Diabetes Mellitus

**DOI:** 10.1155/2019/4902301

**Published:** 2019-05-07

**Authors:** Ying Wang, Shanshan Yang, Qiuyue Guan, Jinglu Chen, Xueping Zhang, Yuwei Zhang, Yiming Yuan, Zhiguang Su

**Affiliations:** ^1^Department of Geriatric Medicine and National Clinical Research Center for Geriatrics, West China Hospital, Sichuan University, Chengdu 610041, China; ^2^Molecular Medicine Research Center, West China Hospital, Sichuan University, Chengdu 610041, China; ^3^Department of Geriatrics, People's Hospital of Sichuan Province, Chengdu, 610041 Sichuan, China; ^4^Division of Endocrinology and Metabolism, West China Hospital, Sichuan University, Chengdu 610041, China

## Abstract

Nuclear factor-Y (NF-Y) consists of three evolutionary conserved subunits including NF-YA, NF-YB, and NF-YC; it is a critical transcriptional regulator of lipid and glucose metabolism and adipokine biosynthesis that are associated with type 2 diabetes mellitus (T2DM) occurrence, while the impacts of genetic variants in the NF-Y gene on the risk of T2DM remain to be investigated. In the present study, we screened five single-nucleotide polymorphisms (SNPs) with the SNaPshot method in 427 patients with T2DM and 408 healthy individuals. Subsequently, we analyzed the relationships between genotypes and haplotypes constructed from these SNPs with T2DM under diverse genetic models. Furthermore, we investigated the allele effects on the quantitative metabolic traits. Of the five tagSNPs, we found that three SNPs (rs2268188, rs6918969, and rs28869187) exhibited nominal significant differences in allelic or genotypic frequency between patients with T2DM and healthy individuals. The minor alleles G, C, and C at rs2268188, rs6918969, and rs28869187, respectively, conferred a higher T2DM risk under a dominant genetic model, and the carriers of these risk alleles (either homozygotes of the minor allele or heterozygotes) had statistically higher levels of fasting plasma glucose, cholesterol, and triglycerides. Haplotype analysis showed that SNPs rs2268188, rs6918969, rs28869187, and rs35105472 formed a haplotype block, and haplotype TTAC was protective against T2DM (OR = 0.76, 95% CI = 0.33-0.82, *P* = 0.004), while haplotype GCCG was associated with an elevated susceptibility to T2DM (OR = 2.33, 95% CI = 1.43-3.57, *P* = 0.001). This study is the first ever observation to our knowledge that indicates the genetic variants of NF-YA might influence a Chinese Han individual's occurrence of T2DM.

## 1. Introduction

Type 2 diabetes mellitus (T2DM), accounting for more than 90% of all cases of diabetes, is increasing rapidly and becoming a major public health threat throughout the world. During the last few decades, the number of people with T2DM has risen to 360 million worldwide, and this is expected to increase up to 592 million by 2035 [1], and this figure is anticipated to increase by 20% in developed countries and by 70% in developing countries in the next 20 years [2]. Many risk factors have been identified to influence the prevalence or incidence of T2DM. In addition to environmental parameters: obesity, dietary habits, physical activity, psychosocial stress, smoking, and so on, the evidence derived from familial studies including those in twins suggests that T2DM has a strong genetic basis [3]. Indeed, numerous studies through either candidate gene approach or the genome-wide association strategies have associated specific genetic variants with T2DM risk. Up to now, more than 88 loci have been identified to confer susceptibility to T2DM [4]. Their effects however are small, which are not enough to explain the heritability of T2DM.

Nuclear factor-Y (NF-Y) is also named CBF that consists of three evolutionary conserved subunits including NF-YA, NF-YB, and NF-YC (also known as CBP-B, CBP-A, and CBP-C, respectively). NF-Y is a ubiquitously expressed protein with CCAAT-binding activity [5]. The CCAAT motif is widely present in promoters of diverse classes of mammalian genes, so its binding partner NF-Y is involved in the regulation of numerous biological processes, such as cell cycle progression [6], embryonic development [7], neurodevelopment [8], cholesterol and fatty acid metabolism [9, 10], and muscle cell differentiation [11]. Recent studies indicate that the altered NF-Y activity is associated with diabetes. G6PC2 (also termed IGRP gene) encodes for the glucose-6-phosphatase catalytic subunit (G6Pase) that is a key gluconeogenic enzyme in hepatocytes; a genetic variant of G6PC2 is found to affect the NF-Y DNA interaction and enhance G6PC2 expression, resulting in an increased hepatic gluconeogenesis and glucose production [12]. We recently found that the NF-YA liver-specific knockout mice showed significantly reduced blood glucose levels; further evidences demonstrated that NF-YA controls glucose production mainly through upregulating the gluconeogenic enzyme expression, such as phosphoenolpyruvate carboxykinase (PEPCK) and G6Pase [13]. Moreover, NF-YA is closely near the T2DM susceptibility locus identified in European and Pakistani descents and Chinese [14]. On the basis of these observations, NF-YA is considered a convincing candidate gene for the predisposition to T2DM.

Owing to the importance of the CCAAT motif in gene transcription and the critical roles of NF-Y in numerous biological processes, there is a significant number of investigations developing agents that alter NF-Y DNA-binding pattern or its activity. Indeed, various compounds have been shown to affect NF-Y activity. Pyrrolobenzodiazepine conjugates are sequence-specific DNA-binding agents affecting NF-Y DNA interactions [15]. The synthetic antitumor agent HNM-176 can suppress the expression of multidrug resistance gene (MDR1) by inhibiting NF-Y activity in cancer cell lines [16]. Histone deacetylase inhibitors, such as trichostatin A, vorinostat, and valproic acid, clearly alter NF-Y activity via the impact on its acetylation [17, 18]. Thus, it is therapeutically feasible to modulate NF-Y activity through designing proper compounds. This strengthens the importance of assessing whether NF-Y may be a risk gene of T2DM. Therefore, this aim of the present study was to examine the relationship between genetic variants in NF-YA gene with a T2DM risk in a Chinese Han population.

## 2. Materials and Methods

### 2.1. Subjects

As described previously [19], a total of 835 unrelated Chinese Han subjects including 427 patients with T2DM and 408 sex- and age-matched healthy individuals were recruited from the West China Hospital of Sichuan University. The anthropometric parameters such as height, weight, and blood pressure were collected. T2DM was diagnosed according to the criteria of 1999 World Health Organization and defining as fasting glucose level ≥ 7.0 mmol/L and/or 2 h postprandial plasma glucose ≥ 11.1 mmol/L. Patients with a history of other forms of diabetes were excluded. The age-matched healthy individuals with normal fasting glucose levels and without family history of diabetes were from physical examination. This study was approved by the ethics review board of West China Hospital, Sichuan University. Written informed consent was obtained from each participant.

### 2.2. Biochemical Measurements

Overnight fasting venous blood was collected using vacutainers and transferred to ethylene diamine tetraacetic acid (EDTA) tubes. After centrifuging at 4000g for 5 min, the plasma was collected for measuring the biochemical parameters. The plasma levels of glucose and lipid profiles (total cholesterol, high-density lipoprotein cholesterol, low-density lipoprotein cholesterol, and triglyceride) were determined with commercially enzymatic kits (Boehringer Mannheim, USA).

### 2.3. SNP Selection and Genotyping

Genotype data of the Chinese population for the NF-YA region were obtained from the 1000 genome project resources (http://www.1000genomes.org/). Five tagSNPs with an *r* ^2^ threshold of 0.8 and a minor allele frequency of 0.05 were selected using Tagger software implemented in Haploview software (Table S1), which are rs2268188, rs6918969, rs28869187, rs35105472, and rs76109475. The genotype of each SNP was determined by the ABI SNaPshot method described previously [20]. Briefly, PCR products (primers are listed in Suppl.  Table 1) containing SNPs were purified by shrimp alkaline phosphatase and exonuclease I and then were mixed with the SNaPshot multiplex and SNaPshot primers (Suppl.  Table 1) for a single base-pair extension. Finally, SNaPshot reaction products were examined on an ABI 3130 Genetic Analyzer (Applied Biosystems, Carlsbad; CA, USA). The data were analyzed by using GeneMapper 4.0 software (Applied Biosystems).

### 2.4. Statistical Analyses

Statistical analyses were performed with SPSS software (SPSS Inc., Chicago, IL, USA). We compared the demographic and biochemical data between cases and controls with Student's *t*-test. We examined the Hardy-Weinberg equilibrium (HWE) of each SNP using two-sided chi-square analysis. The frequences of genotypes or alleles between the T2DM patients and the controls were compared under different genetic models including dominant, recessive, and additive models; Akaike's information criteria (AIC) were used to select the best one with the samllest AIC value. We compared the biochemical parameters between participants with different genotypes at each SNP with a chi-square test. A two-sided significance level of *P* < 0.05 was used for significant tests. Odds ratios (ORs) and its 95% confidence intervals (CIs) were estimated using unconditional logistic regression analyses [21, 22]. Haplotype reconstruction and pairwise linkage disequilibrium (LD) estimation were performed using Haploview 4.2 (https://www.broadinstitute.org/haploview/haploview).

## 3. Results

### 3.1. Characteristics of the Participants

The individual's demographic data and biochemical parameters (Suppl.  Table 2) have been reported previously [19]. Among the 427 participants with T2DM, 219 (51.28%) were male, and 208 (48.72%) were female. Out of 408 control subjects, 209 (51.22%) were male and 199 (48.78%) were female. The mean age of case and control groups was about 58 years. The average BMIs were 24.16 ± 2.25 and 23.28 ± 2.13 in diabetic patients and control individuals, respectively. There is no significant difference regarding gender, age, and BMI between the two groups (*P* > 0.05). Compared to healthy controls, the patients with diabetes had significantly lower plasma HDL-C concentrations (1.22 ± 0.33 mmol/L vs. 1.38 ± 0.37 mmol/L, *P* = 0.008) and statistically higher levels of glucose (9.77 ± 1.15 mmol/L vs. 4.86 ± 0.58 mmol/L, *P* = 0.001), cholesterol (5.11 ± 0.92 mmol/L vs. 4.87 ± 0.88 mmol/L, *P* = 0.009), and triglycerides (1.72 ± 0.53 mmol/L vs. 1.21 ± 0.34 mmol/L, *P* = 0.003), while the LDL-C levels are approximately equal between the two groups (*P* > 0.05). Meanwhile, the systolic and diastolic blood pressures were significantly higher in diabetic patients than those in healthy controls (*P* < 0.01).

### 3.2. Distribution of the NF-YA SNPs between T2DM Patients and Healthy Individuals

**Figure 1:**
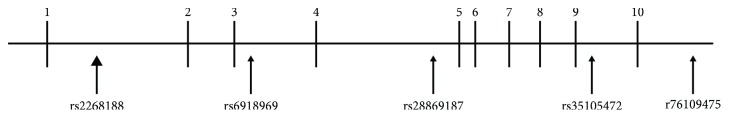
Schematic representation of the NF-YA gene. The relative position of each exon is marked by vertical lines; the arrow indicates the relative location of SNPs genotyped in the current study.

Figure 1 illustrates the physical location of five tagSNPs genotyped. The frequencies of the genotype and allele of each SNP are summarized in Table 1; they were in accordance with the HWE in both groups (*P* > 0.05). We examined the associations of these five tagSNPs with T2DM. Three SNPs (rs2268188, rs6918969, and rs28869187) exhibited nominal significant differences in allelic or genotypic frequencies between patients with T2DM and healthy individuals, whereas others did not. Compared to the allele T at rs2268188, T at rs6918969, and A at rs28869187, the allele G at rs2268188 (OR = 1.31, 95% CI: 1.06-1.61, *P* = 0.011), C at rs6918969 (OR = 1.34, 95% CI: 1.11-1.63, *P* = 0.011), and C at rs28869187 (OR = 1.33, 95% CI: 1.08-1.64, *P* = 0.011) are associated with a higher risk of T2DM, respectively. The genotypes of these three SNPs were distributed statistically differently between T2DM patients and control individuals (*P* < 0.05) (Table 1).

### 3.3. Association of SNPs with T2DM in Diverse Genetic Models

To reach the maximum power in genetic association studies, the genetic model (dominant, recessive, or additive) used in the analysis is required to be concrodant with the “true” inheritant model of disease susceptibility loci. By comparing the frequency distributions of different genotypes at every SNP between groups in diverse models, the best genetic model was determined on the basis of Akaike's information criteria. For each SNP, the allele with relatively lower frequency compared to another one is recognized as the minor allele (Table 1). We found that the minor alleles G, C, and C at rs2268188, rs6918969, and rs28869187, respectively, conferred a significant risk of T2DM under a dominant model (Table 2).

### 3.4. Effects of NF-YA Variants on Metabolic Parameters

**Figure 2:**
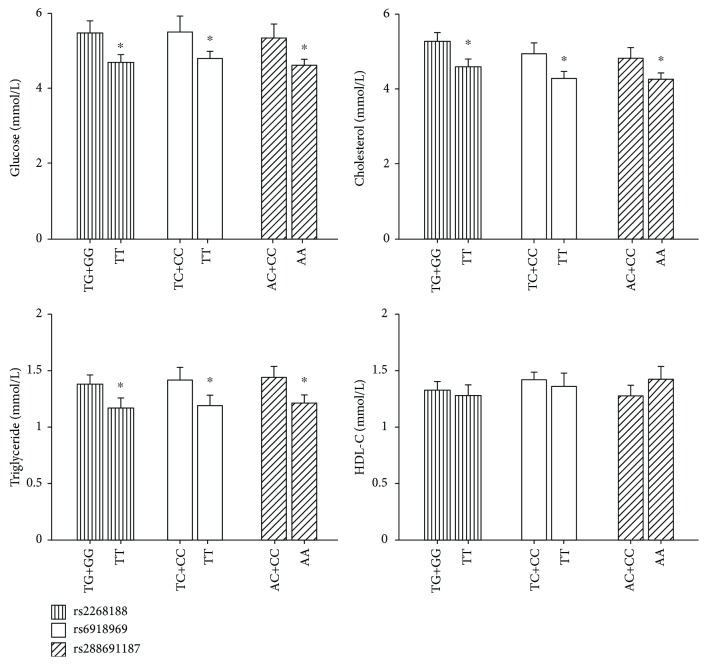
Effects of rs2268188, rs6918969, and rs28869187 on metabolic parameters. The effects were analyzed with covariates gender, age, and body mass index (BMI) in participants with normal glucose regulation under a dominant model. Values are expressed as the means ± SD. ^∗^Statistically significant at *P* < 0.05.

We further examined the effects of rs2268188, rs6918969, and rs28869187 on the metabolic variables in participants with normal glucose regulation under a dominant model. Compared to the individuals with genotypes composed of homozygous wild-type alleles (T, T, and A at rs2268188, rs6918969, and rs28869187, respectively) and the carriers of the risk alleles (either homozygotes of the minor allele or heterozygotes) exhibited statistically higher levels of fasting plasma glucose, cholesterol, and triglycerides (all *P* < 0.05), while no such association was found for HDL-C levels (Figure 2).

### 3.5. NF-YA Haplotypes and T2DM

**Figure 3:**
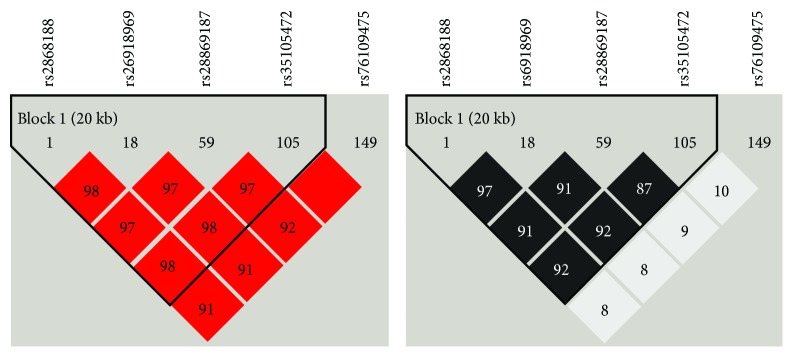
Linkage disequilibrium (LD) plots for SNPs genotyped in the NF-YA region, which were generated by Haploview 4.2 software. SNPs are identified by their rs number in dbSNP. Red shades (left) show the strength of the pairwise LD based on D′ expressed as a percentile within squares. Black shades (right) demonstrate the strength of the pairwise LD based on *r* ^2^, and the number is the *r* ^2^ value expressed as a percentage.

Linkage disequilibrium (LD) in pairwise combinations of alleles in different SNPs was assessed by using maximum likelihood from the genotype frequency in all participants. As shown in Figure 3, four SNPs (rs2268188, rs6918969, rs28869187, and rs35105472) were in moderate LD and formed a haplotype block.

We subsequently compared the frequency distributions of haplotypes constructed from these four SNPs between T2DM patients and controls. As shown in Table 3, haplotype TTAC was associated with a lower risk of T2DM (OR = 0.76, 95% CI = 0.33-0.82, *P* = 0.004), and haplotype GCCG was associated with an elevated susceptibility of T2DM (OR = 2.33, 95% CI = 1.43-3.57, *P* = 0.001). None of the other haplotypes was found to have significant effects.

## 4. Discussion

In this present study, we examined the relationships between five tagSNPs in the NF-YA region and their susceptibilities to T2DM. Our results suggest that the three SNPs (rs2268188, rs6918969, and rs28869187) are associated with the T2DM risk, and their minor alleles could confer an increased T2DM risk under a dominant genetic model. Moreover, the haplotype analysis also strongly supports that the NF-YA genetic variants contribute to the T2DM susceptibility. Haplotypes derived from rs2268188, rs6918969, rs28869187, and rs3510542 were associated with the risk of T2DM, implying the complexity of the NF-YA gene in T2DM development.

Indeed, numerous evidence has shown that NF-Y plays important roles in the regulation of glucose metabolism and the T2DM occurrence. Firstly, NF-Y can activate transcriptions of various endoplasmic reticulum (ER) stress-regulated genes, such as GRP78, XBP-1, ERP72, and CHOP [23, 24]. ER stress has been shown to be an important mediator in the pathogenesis of insulin resistance and diabetes [25]. ER stress induced by excessive glucose or dietary fatty acid triggers pancreatic beta cell apoptosis and insulin resistance in peripheral tissues [26]. Secondly, NF-Y is recently demonstrated to control the expressions of adipokines including adiponectin and leptin in adipose tissue [27, 28]. Over the past decades, emerging evidence has indicated that the content of adipose tissue is associated with insulin resistance and T2DM [29]. Adiponectin and leptin can act as linkers between obesity and insulin resistance by performing special roles in the regulation of body metabolism [30]. Moreover, the adipose tissue-specific NF-Y knockout animals exhibit hyperglycemia and hyperinsulinemia [27]. Finally, NF-Y is identified to control hepatic gluconeogenesis, which is the primary source of glucose production in patients with T2DM. In line with its function in gluconeogenesis, liver-specific NF-YA knockout mice showed significantly decreased fasting blood glucose levels [13]. These observations imply that NF-YA genetic variants might influence the risk of T2DM by combining multiple pathways.

T2DM has been associated with abnormal triglyceride and cholesterol concentrations [31]. In this study, we observed that the diabetic risk alleles were associated with the elevated plasma levels of triglyceride and cholesterol, suggesting the possible participation of genetic variants of NF-YA in T2DM etiology. Indeed, NF-Y is ascertained to regulate the expression of genes encoding the key enzymes responsible for the metabolism of cholesterol and fatty acids, such as 3-hydroxy-3-methylglutaryl-CoA synthase and reductase, squalene synthase, fatty acid synthase, and acetyl-CoA carboxylase [9, 10, 32]. In addition, NF-Y regulates the transcription of SREBP-1[33] that is a master transcription factor of cholesterol and fatty acid biosynthesis [34]. Moreover, the DNA binding of NF-Y predisposes a positive chromatin environment for transcriptional induction by SREBP [35]. Therefore, NF-Y may affect the development of T2DM by regulating the lipid metabolism.

The statistically significantly associated variants are within the NF-YA intron regions, which are considered to have no apparent biological function. However, intronic SNPs might impact gene expression [36]. It is also likely that the diabetic risk variants of NF-YA are only surrogate markers for the causal functional SNPs elsewhere. We only selected SNPs with minor allele frequency of 5% in the present study; thus, those rare disease-causing variants might be missed. However, the rare variants in genetic architecture are demonstrated to be critically important in disease etiology in a recent study [37]. Thus, the investigation of rare variants of NF-YA may lead to a better understanding of their roles in susceptibility to the development of T2DM. In addition, NF-Y is a trimeric protein complex composed by the NF-YA, NF-YB, and NF-YC subunits, which are all necessary for the formation of NF-Y complexes and binding to target gene [5]. The absence of any of the NF-Y subunits results in loss of binding of the NF-Y complex to DNA- and NF-Y-directed transcription [5]. Therefore, it is necessary to establish the association of all three subunits of NF-Y with the diabetes in the future study.

In conclusion, this study is the first ever observation to our knowledge that demonstrates genetic variants of NF-YA are associated with T2DM. It is necessary to determine if the associated alleles influence gene expression, which will clarify the molecular mechanisms underlying the association of NF-Y and T2DM.

## Figures and Tables

**Table 1:** Distributions of the NF-YA SNP in T2DM patients and controls.

SNP	Group	Genotype		HWE^∗^	Allele			
		Number	*P* value	*P* value	Freq. (%)		*P* value	OR [95% CI]^∗∗^
rs2268188		TT/TG/GG			T	G		
	T2DM	191/182/54	**0.013**	0.304	66.0	34.0	**0.011**	1.31 [1.06–1.61]
	Control	214/158/36		0.380	71.8	28.2		
rs6918969		TT/TC/CC			T	C		
	T2DM	144/208/75	**0.005**	0.994	58.1	41.9	**0.004**	1.34 [1.11–1.63]
	Control	179/172/57		0.136	64.9	35.1		
rs28869187		AA/AC/CC			A	C		
	T2DM	182/184/61	**0.008**	0.193	64.2	35.8	**0.006**	1.33 [1.08–1.64]
	Control	208/159/41		0.198	70.5	29.5		
rs35105472		CC/CG/GG			C	G		
	T2DM	218/170/39	0.084	0.482	71.0	29.0	0.081	1.21 [0.97–1.54]
	Control	227/156/25		0.793	74.8	25.2		
rs76109475		GG/GA/AA			G	A		
	T2DM	195/181/51	0.624	0.369	66.9	33.1	0.615	1.05 [0.86–1.29]
	Control	194/167/47		0.231	68.0	32.0		

^∗^HWE: Hardy-Weinberg equilibrium; ^∗∗^OR: odds ratio; CI: confidence interval. *P* values less than 0.05 are in bold.

**Table 2:** Association between NF-YA SNPs and the risk of T2DM under different genetic models.

SNP	Genetic model^∗^		*P* value	OR [95% CI]^∗∗^
rs2268188	Dominant	(TG+GG) vs. TT	**0.026**	**1.36 [1.04–1.79]**
rs6918969	Dominant	(TC+CC) vs. TT	**0.003**	**1.54 [1.16–2.03]**
rs28869187	Dominant	(AC+CC) vs. AA	**0.016**	**1.40 [1.07–1.84]**
rs35105472	Recessive	GG vs. (CG+CC)	0.074	1.62 [0.95–2.77
rs76109475	Dominant	(AG+AA) vs. GG	0.586	1.08 [0.82–1.42]

^∗^For each SNP, only the best genetic model determined by Akaike's information criterion (AIC) is provided. ^∗∗^The ORs and CIs that are statistically significant are bolded, along with the rs number of the corresponding SNP.

**Table 3:** Frequencies of pairwise haplotype constructed by SNPs in NF-YA.

Haplotype^∗^	Frequency		*P* value	OR [95% CI]^∗∗^
	Case	Control		
TTAC	0.337	0.348	**0.004**	**0.76 [0.33–0.82]**
GCCG	0.344	0.319	**0.001**	**2.33 [1.43–3.57]**
GCCC	0.122	0.123	0.954	
TTAG	0.109	0.113	0.443	
CGTG	0.085	0.079	0.217	

^∗^The order of SNPs from left to right is rs2268188, rs6918969, rs28869187, and rs35105472. Only haplotypes with a frequency > 3% in at least one group were listed. ^∗∗^^.^ Only haplotypes distributed significantly differently (*P* < 0.05) were calculated.

## Data Availability

Data are included in this published article and its supplementary information files. We can provide detailed data upon reasonable request.

## Supplementary Materials

Supplementary Materials Supplemental Table 1. Five SNPs in the NF-YA gene in the study. Supplemental Table 2. Characteristics of T2DM patients and control subjects.

## References

[1] Guariguata L., Whiting D. R., Hambleton I., Beagley J., Linnenkamp U., Shaw J. E. (2014). Global estimates of diabetes prevalence for 2013 and projections for 2035. *Diabetes Research and Clinical Practice*.

[2] Chen L., Magliano D. J., Zimmet P. Z. (2012). The worldwide epidemiology of type 2 diabetes mellitus—present and future perspectives. *Nature Reviews Endocrinology*.

[3] Stumvoll M., Goldstein B. J., van Haeften T. W. (2008). Type 2 diabetes: pathogenesis and treatment. *Lancet*.

[4] Mohlke K. L., Boehnke M. (2015). Recent advances in understanding the genetic architecture of type 2 diabetes. *Human Molecular Genetics*.

[5] Dolfini D., Gatta R., Mantovani R. (2012). NF-Y and the transcriptional activation of CCAAT promoters. *Critical Reviews in Biochemistry and Molecular Biology*.

[6] Bungartz G., Land H., Scadden D. T., Emerson S. G. (2012). NF-Y is necessary for hematopoietic stem cell proliferation and survival. *Blood*.

[7] Bhattacharya A., Deng J. M., Zhang Z., Behringer R., de Crombrugghe B., Maity S. N. (2003). The B subunit of the CCAAT box binding transcription factor complex (CBF/NF-Y) is essential for early mouse development and cell proliferation. *Cancer Research*.

[8] Yamanaka T., Tosaki A., Kurosawa M. (2014). NF-Y inactivation causes atypical neurodegeneration characterized by ubiquitin and p62 accumulation and endoplasmic reticulum disorganization. *Nature Communications*.

[9] Inoue J., Sato R., Maeda M. (1998). Multiple DNA elements for sterol regulatory element-binding protein and NF-Y are responsible for sterol-regulated transcription of the genes for human 3-hydroxy-3-methylglutaryl coenzyme A synthase and squalene synthase. *Journal of Biochemistry*.

[10] Xiong S. B., Chirala S. S., Wakil S. J. (2000). Sterol regulation of human fatty acid synthase promoter I requires nuclear factor-Y- and Sp-1-binding sites. *Proceedings of the National Academy of Sciences*.

[11] Gurtner A., Manni I., Fuschi P. (2003). Requirement for down-regulation of the CCAAT-binding activity of the NF-Y transcription factor during skeletal muscle differentiation. *Molecular Biology of the Cell*.

[12] Bouatia-Naji N., Bonnefond A., Baerenwald D. A. (2010). Genetic and functional assessment of the role of the rs13431652-A and rs573225-A alleles in the G6PC2 promoter that are strongly associated with elevated fasting glucose levels. *Diabetes*.

[13] Zhang Y., Guan Q., Liu Y. (2018). Regulation of hepatic gluconeogenesis by nuclear factor Y transcription factor in mice. *Journal of Biological Chemistry*.

[14] Gan W., on behalf of the China Kadoorie Biobank Collaborative Group, Walters R. G. (2016). Evaluation of type 2 diabetes genetic risk variants in Chinese adults: findings from 93,000 individuals from the China Kadoorie Biobank. *Diabetologia*.

[15] Henry J. A., Le N. M., Nguyen B. (2004). Targeting the Inverted CCAAT Box 2 in the Topoisomerase II*α* Promoter byJH-37, an Imidazole−Pyrrole Polyamide Hairpin: Design, Synthesis, Molecular Biology, and Biophysical Studies†. *Biochemistry*.

[16] Tanaka H., Ohshima N., Ikenoya M., Komori K., Katoh F., Hidaka H. (2003). HMN-176, an active metabolite of the synthetic antitumor agent HMN-214, restores chemosensitivity to multidrug-resistant cells by targeting the transcription factor NF-Y. *Cancer Research*.

[17] Campanero M. R., Herrero A., Calvo V. (2008). The histone deacetylase inhibitor trichostatin A induces GADD45 gamma expression via Oct and NF-Y binding sites. *Oncogene*.

[18] Huang W. Q., Zhao S. J., Ammanamanchi S., Brattain M., Venkatasubbarao K., Freeman J. W. (2005). Trichostatin A Induces Transforming Growth Factor *β* Type II Receptor Promoter Activity and Acetylation of Sp1 by Recruitment of PCAF/p300 to a Sp1·NF-Y Complex. *Journal of Biological Chemistry*.

[19] Zhang Y., Liu Y., Liu Y., Zhang Y., Su Z. (2016). Genetic variants of retinoic acid receptor-related orphan receptor alpha determine susceptibility to type 2 diabetes mellitus in Han Chinese. *Genes*.

[20] Yuan Y., Jiang H., Kuang J., Hou X., Feng Y., Su Z. (2012). Genetic variations in ADIPOQ gene are associated with chronic obstructive pulmonary disease. *PloS One*.

[21] Altman D. G., Bland J. M. (2011). How to obtain the confidence interval from a P value. *BMJ*.

[22] Bland J. M., Altman D. G. (2000). Statistics Notes: The odds ratio. *BMJ*.

[23] Yoshida H., Okada T., Haze K. (2000). ATF6 activated by proteolysis binds in the presence of NF-Y (CBF) directly to the cis-acting element responsible for the mammalian unfolded protein response. *Molecular and Cellular Biology*.

[24] Liu Y., Zhang Y., Zhang Y. (2017). Obesity-induced endoplasmic reticulum stress suppresses nuclear factor-Y expression. *Molecular and Cellular Biochemistry*.

[25] Ghemrawi R., Battaglia-Hsu S.-F., Arnold C. (2018). Endoplasmic reticulum stress in metabolic disorders. *Cells*.

[26] Gurlo T., Rivera J. F., Butler A. E. (2016). CHOP Contributes to, But Is Not the Only Mediator of, IAPP Induced *β*-Cell Apoptosis. *Molecular Endocrinology*.

[27] Lu Y. H., Dallner O. S., Birsoy K., Fayzikhodjaeva G., Friedman J. M. (2015). Nuclear factor-Y is an adipogenic factor that regulates leptin gene expression. *Molecular Metabolism*.

[28] Park S. K., Oh S. Y., Lee M. Y., Yoon S., Kim K. S., Kim J. W. (2004). CCAAT/enhancer binding protein and nuclear factor-Y regulate adiponectin gene expression in adipose tissue. *Diabetes*.

[29] Czech M. P. (2017). Insulin action and resistance in obesity and type 2 diabetes. *Nature Medicine*.

[30] Jaganathan R., Ravindran R., Dhanasekaran S. (2018). Emerging role of adipocytokines in type 2 diabetes as mediators of insulin resistance and cardiovascular disease. *Canadian Journal of Diabetes*.

[31] Tomkin G. H., Owens D. (2017). Diabetes and dyslipidemia: characterizing lipoprotein metabolism. *Diabetes, Metabolic Syndrome and Obesity: Targets and Therapy*.

[32] Teran-Garcia M., Rufo C., Nakamura M. T., Osborne T. F., Clarke S. D. (2002). NF-Y involvement in the polyunsaturated fat inhibition of fatty acid synthase gene transcription. *Biochemical and Biophysical Research Communications*.

[33] Amemiya-Kudo M., Shimano H., Yoshikawa T. (2000). Promoter analysis of the mouse sterol regulatory element-binding protein-1c gene. *Journal of Biological Chemistry*.

[34] Jeon T. I., Osborne T. F. (2012). SREBPs: metabolic integrators in physiology and metabolism. *Trends in Endocrinology & Metabolism*.

[35] Reed B. D., Charos A. E., Szekely A. M., Weissman S. M., Snyder M. (2008). Genome-wide occupancy of SREBP1 and its partners NFY and SP1 reveals novel functional roles and combinatorial regulation of distinct classes of genes. *PLoS Genet*.

[36] Korb M., Ke Y., Johnson L. F. (1993). Stimulation of gene expression by introns: conversion of an inhibitory intron to a stimulatory intron by alteration of the splice donor sequence. *Nucleic Acids Research*.

[37] Nelson M. R., Wegmann D., Ehm M. G. (2012). An abundance of rare functional variants in 202 drug target genes sequenced in 14,002 people. *Science*.

