# Supplementary material for: Effects of Genetic Variants of Nuclear Receptor Y on the Risk of Type 2 Diabetes Mellitus
Source: J Diabetes Res. 2019 May 7;2019:4902301. doi: 10.1155/2019/4902301 (PMC6530108; doi:10.1155/2019/4902301)
Supplement: Supplementary Materials — Supplemental Table 1. Five SNPs in the NF-YA gene in the study. Supplemental Table 2. Characteristics of T2DM patients and control subjects. [file 4902301.f1.docx]

**Supplemental Table 1.** Five SNPs in the NF-YA gene in the study

| SNPs | Position  (GRCh37.p13) | Alleles | Primers (5’-3’) | |
| --- | --- | --- | --- | --- |
|  |  |  | PCR | SNaPshot |
| rs2268188 | 41042590 | T/G | GCACCAGATACTTAAGAC | CTAGGACAAGTCTCCTCTTC |
|  |  |  | GGAGGTTCAAGTAGAAGG |  |
| rs6918969 | 41049895 | T/C | CCTCCATTGAATTAGAAATG | ATCCAGCAGATAACTACCTCTTA |
|  |  |  | GAATGCCTACAAGCTCAG |  |
| rs28869187 | 41056505 | A/C | CTCAAATGATCCTCCCACC | ATGCCT GGATAATTTTTGTATTTTTC |
|  |  |  | GCTGAAAGCCGGGTAC |  |
| rs35105472 | 41062689 | C/G | GTTGAGATGCTCT TCGTC | CCATGCCTGGATAATTTTTGTATTTTTC |
|  |  |  | AAGTGGAAGAAGGGAAACC |  |
| rs76109475 | 41069120 | G/A | GAGCTAAGGCACAGTTTAAC | TTATTGAACAGGTTTTATTACC |
|  |  |  | GAACGCTTAATAGAAAATGC |  |

**Supplemental Table 2.** Characteristics of T2DM patients and control subjects **^a^**

| **Variable** | **Cases (n = 427)** | **Controls (n = 408)** | **p** |
| --- | --- | --- | --- |
| Age (years) | 57.37 ± 11.28 | 58.26 ± 10.51 | 0.062 ^b^ |
| Sex (Men/Women) | 219/208 | 209/199 | 0.075 ^b^ |
| BMI (kg/m^2^) ^c^ | 24.16 ± 2.25 | 23.28 ± 2.13 | 0.086 ^b^ |
| SBP (mmHg) ^c^ | 140.16 ± 18.21 | 124.41 ± 14.18 | **0.007** ^d^ |
| DBP (mmHg) ^c^ | 83.47 ± 8.68 | 77.63 ± 9.32 | **0.008** ^d^ |
| FPG (mmol/L) ^c^ | 9.77 ± 1.15 | 4.86 ± 0.58 | **0.001** ^d^ |
| TC (mmol/L) ^c^ | 5.11 ± 0.92 | 4.87 ± 0.88 | **0.009** ^d^ |
| HDL-C (mmol/L) ^c^ | 1.22 ± 0.33 | 1.38 ± 0.37 | **0.008** ^d^ |
| LDL-C (mmol/L) ^c^ | 2.77 ± 0.82 | 2.69 ± 0.91 | 0.073 ^b^ |
| TG (mmol/L) ^c^ | 1.72 ± 0.53 | 1.21 ± 0.34 | **0.003** ^d^ |

^a^Data are presented as mean ± standard deviation (SD). ^b^ no significant difference (*P*> 0.05). ^c^ BMI, body mass index, which is calculated as weight in kilograms divided by height in meters squared; SBP, systolic blood pressure; DBP, diastolic blood pressure; FPG, fasting plasma glucose. TC: total cholesterol; HDL: high-density lipoprotein; LDL: low-density lipoprotein; TG: triglyceride. ^d^ Statistically significant at *P* < 0.05 in bold.
